# The healthy beverage index is not associated with insulin resistance, prediabetes and type 2 diabetes risk in the Rotterdam Study

**DOI:** 10.1007/s00394-023-03209-6

**Published:** 2023-07-25

**Authors:** Maria G. Jacobo Cejudo, Carolina Ochoa-Rosales, Fariba Ahmadizar, Maryam Kavousi, Johanna M. Geleijnse, Trudy Voortman

**Affiliations:** 1 Division of Human Nutrition and Health, Wageningen University, PO Box 17, 6700 AA Wageningen, The Netherlands; 2 Department of Epidemiology, Erasmus MC, University Medical Center Rotterdam, Rotterdam, The Netherlands; 3 Latin American Brain Health Institute (BrainLat), Universidad Adolfo Ibáñez, Santiago, Chile; 4 Department of Data Science & Biostatistics, Julius Global Health, University Medical Center Utrecht, Utrecht, The Netherlands

**Keywords:** Healthy beverage index, Beverage quality, Repeated measurements of HOMA-IR, Prediabetes incidence, Type 2 diabetes incidence

## Abstract

**Purpose:**

Whether beverage quality affects changes in glycaemic traits and type 2 diabetes (T2D) risk is unknown. We examined associations of a previously developed Healthy Beverage Index (HBI) with insulin resistance, and risk of prediabetes and T2D.

**Methods:**

We included 6769 participants (59% female, 62.0 ± 7.8 years) from the Rotterdam Study cohort free of diabetes at baseline. Diet was assessed using food-frequency questionnaires at baseline. The HBI included 10 components (energy from beverages, meeting fluid requirements, water, coffee and tea, low-fat milk, diet drinks, juices, alcohol, full-fat milk, and sugar-sweetened beverages), with a total score ranging from 0 to 100. A higher score represents a healthier beverage pattern. Data on study outcomes were available from 1993 to 2015. Multivariable linear mixed models and Cox proportional-hazards regression models were used to examine associations of the HBI (per 10 points increment) with two measurements of HOMA-IR (a proxy for insulin resistance), and risk of prediabetes and T2D.

**Results:**

During follow-up, we documented 1139 prediabetes and 784 T2D cases. Mean ± SD of the HBI was 66.8 ± 14.4. Higher HBI score was not associated with HOMA-IR (β: 0.003; 95% CI − 0.007, 0.014), or with risk of prediabetes (HR: 1.01; 95% CI 0.97, 1.06), or T2D (HR: 1.01; 95% CI 0.96, 1.07).

**Conclusion:**

Our findings suggest no major role for overall beverage intake quality assessed with the HBI in insulin resistance, prediabetes and T2D incidence. The HBI may not be an adequate tool to assess beverage intake quality in our population.

**Supplementary Information:**

The online version contains supplementary material available at 10.1007/s00394-023-03209-6.

## Introduction

Diet constitutes an important modifiable lifestyle factor in the prevention and management of type 2 diabetes (T2D) [1]. Besides, beverages constitute an important component of the diet because they contribute to fulfill our daily fluid requirements, and they can contribute to the overall nutrient and energy intakes [2–4]. Commonly consumed beverages are water, coffee, tea, milk, fruit juices, sugar-sweetened beverages (SSBs), and alcoholic beverages [3].

Given the diverse nutritional composition of beverages, they have been differentially associated with cardiometabolic outcomes. SSBs are major contributors to energy and added sugars to the diet and have been consistently associated to higher risk of obesity, T2D, and other cardiometabolic diseases [5–7]. In line with this, replacing SSB with water has been associated with a lower risk of these diseases [8–10]. Fruit juices may contain as much sugar and calories as SSBs [11, 12], and have also been associated with increased risk of T2D [5] and weight gain [12]. Recent evidence has suggested that long-term consumption of artificially sweetened beverages may also be associated to increased risk of T2D [5, 13]. Both, coffee and tea have been related to lower risk of T2D and other cardiometabolic diseases [14–19]. Recent scientific evidence has shown neutral to modest inverse associations for low-fat milk consumption with T2D risk [20, 21], whereas whole-fat milk may lead to higher cardiometabolic risk because of increased risk of weight gain [22, 23]. Finally, low and moderate alcohol consumption has been associated to lower risk of T2D, but also increased risk of stroke, coronary artery disease (except MI) and hypertension [24, 25].

Although several beverage groups seem to influence cardiometabolic risk, to date few studies have investigated overall beverage intake quality and cardiometabolic outcomes, by using a priori developed scoring systems [26–28]. The healthy beverage index (HBI) developed by Duffey and Davy [26], is a scoring system that includes scores for recommended intakes of various beverage groups, total fluid consumption, and energy from beverages to evaluate overall beverage intake quality. A higher score in the HBI representing a healthier beverage intake pattern was cross-sectionally associated with lower cardiometabolic risk in a representative sample of the US population, as reflected by lower odds ratios of having hypertension, high fasting insulin levels, and high low-density lipoprotein cholesterol level. However, the longitudinal association between overall beverage intake quality and T2D risk has not been explored. Besides, for public health advice, it is of interest to know if a healthier beverage intake pattern may also influence T2D since early stages (insulin resistance and prediabetes). Therefore, we aimed to investigate the association of HBI score with insulin resistance, prediabetes risk and T2D risk in an adult Dutch population.

## Methods

### Study design and population

This study was carried-out in three sub-cohorts of the Rotterdam Study (RS). The RS is an ongoing prospective, population-based cohort study on risk factors for chronic diseases in mid and late life. Its design has been described elsewhere [29]. Briefly, the first sub-cohort (RS-I) started in 1990–1993, with the inclusion of 7983 inhabitants of the Ommoord district in the Dutch city of Rotterdam, that were 55 years or over. In 2000–2001, the second sub-cohort (RS-II) was initiated with the inclusion of 3011 new subjects who became 55 years old or moved to the study district. In 2006, the third sub-cohort (RS-III) was initiated with the inclusion of 3932 individuals aged 45 years or over. By the end of 2008, the Rotterdam Study comprised 14,926 subjects aged 45 years or over. At baseline, participants were interviewed at home and then extensively examined in a study research center. Follow-up examinations were performed every 3–5 years in each sub-cohort.

For the current study, baseline data from sub-cohorts RS-I, RS-II, and RS-III were used (n = 14,926). We excluded participants with missing data on diet (n = 5176), implausible estimated daily energy intake (cut-offs set at < 500 or > 5000 kcal/day, as previously done in the RS; n = 50), prevalent T2D at baseline (n = 963), or missing data on prevalent T2D (n = 1968), resulting in 6769 participants. For the analysis on insulin resistance, of the 6769 participants, we additionally excluded n = 2873 subjects who did not have at least two measurements of HOMA-IR, one at baseline and one at follow-up, resulting in 3896 participants. For the analysis on pre-diabetes risk, from 6769 participants, we additionally excluded subjects with prediabetes at baseline or without follow-up data on prediabetes (n = 717), resulting in 6052 participants. Finally, for the analysis of T2D, from the sub-sample of 6769, we additionally excluded subjects with missing data on follow-up T2D (n = 51), resulting in 6718 participants (Supplementary Fig. 1).

The Rotterdam Study has been approved by the Medical Ethics Committee of the Erasmus MC (registration number MEC 02.1015) and by the Dutch Ministry of Health, Welfare and Sport (Population Screening Act WBO, license number 1071272-159521-PG). The Rotterdam Study Personal Registration Data collection is filed with the Erasmus MC Data Protection Officer under registration number EMC1712001. The Rotterdam Study has been entered into the Netherlands National Trial Register (NTR; www.trialregister.nl) and into the World Health Organization (WHO) International Clinical Trials Registry Platform (ICTRP; www.who.int/ictrp/network/primary/en/) under shared catalogue number NTR6831. All subjects gave written informed consent, to participate in the study and to have their information obtained from treating physicians.

### Assessment of dietary intake

At baseline, dietary intake data were collected using validated semi-quantitative food-frequency questionnaires (FFQs). A 170-item FFQ was used for sub-cohorts RS-I and RS-II, and a 389-item FFQ was used for RS-III. The preceding year served as the reference period for RS-I and RS-II, and the last month for RS-III. Details are provided elsewhere [30]. Both FFQs were previously validated for nutrient intakes against other dietary assessment methods, and demonstrated to adequately rank participants according to their intake [30–32]. Food and beverage intake data were converted to nutrient and energy data through linkage with the Dutch Food-Composition Table corresponding to the year the dietary evaluation was performed (1993 for RS-I, 2001 for RS-II and 2011 for RS-III).

### Calculation of HBI components and scores

Based on the HBI developed by Duffey and Davy [26], 10 components were created (Table 1). Briefly, overall daily beverage intake was grouped according to the US Beverage Guidance System [2] into eight groups (components): water, coffee and tea, low-fat and skimmed milk (< 2.0% fat), diet beverages (non-calorically sweetened beverages, except for RS-I), fruit and vegetable juices, alcoholic beverages (including beer, wine and liquors), whole-fat milk (≥ 2.0% fat), and SSBs (Supplementary Table 1). Beverage intake was converted from mL/day to percentage of intake according to total fluid requirements, except for alcoholic beverages, which were assessed as glasses of alcohol consumed per day. In the Netherlands, a standard glass of alcohol contains 10 g of ethanol. Two additional components were included, total energy from beverages and meeting total fluid requirements defined as 1 mL of fluid per 1 kcal of food consumed [26].

**Table 1:** Components of the healthy beverage index (HBI) and their scoring system as applied in the current analysis [26]^a^

Component	Description	Scores
Water	- ≥ 20% of fluid requirements - No water consumption - < 20% of fluid requirements	15 0 Proportional points between > 0 and < 15 based on reported intake^c^
Coffee and tea	Unsweetened coffee and tea: 0–40% of fluid requirements	5
Low-fat milk	< 2.0% fat or fat-free milk: 0–16% of fluid requirements	5
Diet beverages	Artificially-sweetened beverages: 0–16% of fluid requirements	5
Fruit and vegetable juices	Fruit and vegetable juices: 0–8% of fluid requirements	5
Alcohol	From 0–1 standard glass/day for women and from 0 to 2 standard glasses/day for men^b^	5
Full-fat milk	0% of fluid requirements coming from ≥ 2.0% fat milk, sweetened milks, and soy beverages	5
Sugar-sweetened beverages	0–8% of fluid requirements coming from sugar-sweetened beverages, fruit drinks, and other sweetened beverages	15
Total energy from beverages	Energy from beverages < 10% of total energy intake Energy from beverages ≥ 10% but < 15% of total energy intake Energy from beverages ≥ 15% of total energy intake	20 Proportional points between > 0 and < 20 based on reported intake^c^ 0
Meeting total fluid requirements	Amount of beverages (mL) consumed ≥ to fluid requirements Amount of beverages (mL) consumed < than fluid requirements	20 Proportional points between > 0 and < 20 based on reported intake^c^

^a^Modified version based on Duffey and Davy. Compared to the original HBI paper, soy beverages were not included in milk components because of their different nutritional composition, and because in the Netherlands they are not fortified. Herbal infusions were also not included in coffee and tea component, because most of the health benefits of tea intake have been attributed to green and black tea. However, soy beverages and herbal teas were taken into account for the calculation of the scores for the components energy from beverages and meeting total fluid requirements

^b^One alcoholic drink was defined as 10 g of ethanol a day instead of ~ 14 g/day, as this is the standard measure in the Netherlands

^c^Proportional scores were assigned as follows: proportional points for water = [(mL of water × 15)/(0.20 × total fluid requirements)]; proportional points for total energy from beverages = [(15–percentage of energy from beverages) × 10/3] when energy from beverages is between 10 and 14%; proportional points for meeting total fluid requirements = [(total fluids consumed/total fluid requirements) × 20] where total fluid requirements = 1 mL per 1 kcal of food consumed

Total score on the HBI is the sum of the individual component scores, and it ranges from 0 to 100, with a higher score indicating better adherence to beverage guidelines and, thus, a healthier beverage intake pattern. For most components, participants received the maximum score per component if they adhered to the recommended intakes, otherwise they received 0 points (dichotomous scoring system) [26]. Only the components water, energy from beverages and meeting total fluid requirements received proportional points according to reported intake (continuous scoring system). The maximum score possible was higher for those components representing aspects of beverage intake that are more critical to health, such as water and SSBs (15 points), and the additional components energy from beverages and meeting total fluid requirements (20 points).

### Assessment of insulin resistance

Homeostatic model assessment of insulin resistance (HOMA-IR) was calculated using the formula: $fasting insulin \left( \frac{mU}{L} \right)x fasting glucose \left( \frac{mmol}{L} \right)\frac{{}}{{}}22.5$. Fasting blood was drawn at the research center at two examination visits in each sub-cohort: at RS-I-3 (1997–99) and RS-I-5 (2009–11), at RS-II-1 (2000–01) and RS-II-3 (2011–12), and at RS-III-1 (2006–08) and RS-III-2 (2012–14). Because no fasting blood samples were collected in the first two visits of RS-I, we set the third visit (RS-I-3) as the baseline. Glucose levels were measured using the glucose hexokinase method. Serum insulin levels were measured by radioimmunoassay (Medgenix diagnostics, Brussels, Belgium) on a Roche Modular Analytics E170 analyzer.

### Assessment of prediabetes and type 2 diabetes

Prediabetes and T2D cases at baseline and during follow-up examinations were ascertained based on general practitioners’ records, hospital discharge letters, and blood glucose measurements. Prediabetes and T2D were defined according to the WHO guidelines [33]. Diabetes was defined as fasting plasma glucose concentration ≥ 7.0 mmol/L, non-fasting plasma glucose concentration ≥ 11.1 mmol/L when fasting glucose was not available, or use of blood glucose-lowering medication (ATC code A010) [34, 35]. Information about blood glucose-lowering medication use was obtained from structured home interviews and pharmacy dispensing records. Prediabetes was defined as having fasting plasma glucose > 6.0 and < 7.0 mmol/L or non-fasting plasma glucose > 7.7 and < 11.1 mmol/L. Two study physicians independently identified all potential incident prediabetes and T2D cases. In case of disagreement, a consensus was sought by consulting diabetologists [36]. Data of incident prediabetes and T2D was collected until 01-01-2015.

### Assessment of covariates

At baseline and during home interviews, trained research assistants obtained information on health status, smoking behaviour, and socioeconomic status. Self-reported smoking status was classified into three categories (never, former, or current). Educational attainment was self-reported and classified into: primary education with or without a partially completed higher education (primary); lower vocational or lower secondary education (lower); intermediate vocational education and or general secondary (intermediate); or higher vocational or university education (higher). Physical activity was assessed with an adapted version of the Zutphen Physical Activity Questionnaire at RS-I-3 and RS-II-1 [37], and with the LASA Physical Activity Questionnaire at RS-III-1 [38]. Physical activities were weighted according to the intensity with Metabolic Equivalent of Task (MET), from the Compendium of Physical Activities version 2011. To account for differences between the two questionnaires, questionnaire-specific z-scores of MET-hours per week were calculated. Overall diet quality was assessed with adherence (yes/no) to 14 items of the Dutch dietary guidelines 2015: vegetables, fruit, whole-grains, legumes, nuts, dairy, fish, tea, ratio whole-grains:total grains, ratio unsaturated fats and oils:total fats, red and processed meat, sugar-containing beverages, alcohol and salt, with a total score ranging from 0 to 14 [30]. Information on medical history and medication was obtained from interviews, medical records and pharmacy records.

Physical examinations were held at the research center. Height (meters, m) and weight (kilograms, kg) were measured while wearing indoor clothes without shoes. Body mass index (BMI) was calculated as weight divided by height squared (kg/m^2^), and then categorized into normal weight, overweight and obese according to the WHO standards [39].

### Statistical analysis

Descriptive data were presented as mean ± SD for continuous variables with a normal distribution, as median (IQR) for continuous variables with a skewed distribution, or in absolute and relative numbers (percentages) for categorical variables. Median (IQR) scores of the 10 components of the HBI are presented across tertiles of total HBI score to identify which components differentiated healthier from less healthy HBI scores (Supplementary Table 2).

We used linear mixed-effect models (LMM) with a random-effects structure including a random intercept and slope (for participants and time of repeated measurements, respectively) to examine associations between the HBI score (per 10 points increment) and HOMA-IR over time in participants for whom at least two measurements of HOMA-IR were available. HOMA-IR values were natural log-transformed to improve distribution of the residuals (Supplementary Figs. 2 and 3), and results were presented as regression coefficients (β) with their corresponding 95% confidence intervals (CI). We used multivariable Cox proportional hazard models to analyse associations between the HBI score (per 10 points increment) and incident prediabetes and T2D. The proportional hazards assumption was confirmed visually using a log-minus-log plot of the survival time. Person-years were calculated from the time of entering the Rotterdam Study to date of prediabetes or T2D incidence, death, withdrawal from the study or end of follow-up (January 1, 2015), whichever came first. Results were presented as hazard ratios (HR) with their corresponding 95% CI. Main analyses were performed for the overall study population (pooled data of the three RS sub-cohorts). Results by sub-cohort are shown in supplementary materials. Non-linearity of the associations was explored using natural cubic splines (degrees of freedom = 3). As no indications for non-linear associations for the main models were found (p ≥ 0.05, Supplementary Figs. 4 and 5), all primary analyses were performed assuming linearity. Multivariable models included potential confounders and established risk factors for the outcomes. Model 1 was adjusted for baseline age (years), sex, total energy intake (kcal/day), Rotterdam Study sub-cohort (only for overall study population analyses), and time of repeated measurements of HOMA-IR (only for HOMA-IR analyses). Model 2 was additionally adjusted for smoking status, educational attainment, physical activity level and diet quality score. Finally, model 3 was additionally adjusted for baseline BMI (kg/m^2^) as a potential confounder in the associations. Effect modification was examined by including interactions of the HBI score with age, sex, and BMI (continuous and in categories) for all outcomes in model 3.

Additionally, we examined associations between individual components of the HBI and the study outcomes, by treating the component scores as dichotomous variables (adherence vs. non-adherence or partial adherence to the component criteria). We used model 3 additionally adjusting for all other components of the HBI, except for water analyses, which were not additionally adjusted for meeting total fluid requirements because of collinearity issues. Sub-cohort RS-I was excluded from the analyses of diet beverages due to lacking data on diet beverages intake in that sub-cohort.

We performed several sensitivity analyses based on model 3 to examine the robustness of our findings. First, we repeated our main analyses using different variations of the HBI. We excluded scores of the additional components ‘total energy from beverages’ and ‘meeting total fluid requirements’ from the total HBI score (alternative score 1), to see the effect of these components in our associations, because they were the most heavily weighted, additionally adjusting for the excluded components. Second, we repeated main analyses additionally excluding water component score from alternative score 1, because of limited detailed data about water consumption (alternative score 2) and additionally adjusting for the excluded components. Third, to test if the associations were driven by specific components of the HBI, we repeated main analyses by excluding each of the 10 components from the total HBI score one at a time, and adjusting for the excluded component. Fourth, we repeated main analyses assessing the HBI score in tertiles. Fifth, we excluded participants with prevalent cardiovascular diseases (CVDs) at baseline because they are closely interrelated with glucose/metabolic traits and diet. Last, to address possible reverse causation, we excluded participants that developed prediabetes and T2D within the first 3 years of follow-up.

Missing values in covariates (ranging from 0.2 to 3.9%) were imputed with tenfold multiple imputation with chained equations. The results shown correspond to the pooled results of the 10 imputed datasets. Total score in the HBI and individual components scores were calculated using SPSS statistical software, version 21.0 (IBM Corp, Armonk, NY). All other statistical analyses were performed using R version 3.3.1 (The R Foundation for Statistical Computing, Vienna, Austria). A two-sided p-value < 0.05 was considered statistically significant.

## Results

### Baseline characteristics

Baseline characteristics in the overall study population are shown in Table 2 and by RS sub-cohort in Supplementary Table 3. In our population of 6769 participants, the mean ± SD age was 62.0 ± 7.8 years and 59% were female. The three most consumed beverage groups were [median (IQR) in consumers]: coffee and tea [750 (550, 1000) mL/day], water [349 (175, 611) mL/day] and low-fat milk [224 (139, 352) mL/day]. Total score on the HBI (with a theoretical range from 0 to 100) ranged from 22.4 to 100, with a mean ± SD of 66.8 ± 14.4 (Table 2). The three components to which participants adhered the most to recommended intakes were diet drinks (98% adherence), SSBs (93% adherence), and fruit and vegetable juices (87% adherence) (Supplementary Table 4).

**Table 2:** Baseline characteristics in the overall study population (n = 6769)^a^

Age (years)	62.0 ± 7.8
Sex, n (% female)	3977 (59%)
Smoking, n (%)	
Never	2205 (32%)
Former	3146 (47%)
Current	1418 (21%)
Educational level, n (%)	
Primary	794 (12%)
Lower	2752 (41%)
Intermediate	1947 (29%)
Higher	1233 (18%)
Physical activity (METh/w), RS-I and II^b^	79.7 (54.7, 112.1)
Physical activity (METh/w), RS-III^b^	42.9 (17.7, 82.5)
BMI, (kg/m^2^)	26.7 ± 4.8
Plasma glucose (mmol/L)^c^	5.40 (5.10, 5.80)
Serum insulin (pmol/L)^c^	69.0 (49.0, 97.0)
HOMA-IR^c^	3.40 ± 2.81
Dietary intake	
Total energy (kcal/day)	2144 ± 616
Energy from beverages (kcal/day)	232 (143, 347)
Water, n (%) of consumers	4724 (70%)
mL/day only in consumers	349 (175, 611)
Coffee and tea, n (%) of consumers	6707 (99%)
mL/day only in consumers	750 (550, 1000)
Low-fat milk, n (%) of consumers	5252 (78%)
mL/day only in consumers	224 (139, 352)
Diet beverages, n (%) of consumers	905 (24%)
mL/day only in consumers^d^	64 (23, 175)
Juices, n (%) of consumers	4,271 (63%)
mL/day only in consumers	71 (27, 140)
Alcohol, n (%) of consumers	5676 (84%)
glasses/day only in consumers^e^	1.00 (0.27, 2.15)
Full-fat milk, n (%) of consumers	2787 (41%)
mL/day only in consumers	32 (16, 80)
Sugar-sweetened beverages, n (%) of consumers	2592 (38%)
mL/day only in consumers	50 (21, 142)
Total beverage (mL/day)	1538 (1211, 1952)
Total HBI score	66.8 ± 14.4
Diet quality score^f^	6.8 ± 1.9

*HBI* healthy beverage index, *HOMA-IR* Homeostatic model assessment of insulin resistance, *IQR* interquartile range, *RS* Rotterdam Study, *SD* standard deviation

^a^Values are mean ± SD for continuous variables with normal distribution, median (IQR) for continuous variables with a skewed distribution or absolute numbers with (percentages) for categorical variables based on non-imputed data. Missing data for variables was as follows: n = 43 for educational level, n = 265 for physical activity, n = 87 for BMI, n = 151 for blood glucose, n = 307 for serum insulin, and n = 331 for HOMA-IR

^b^Zutphen Physical Activity questionnaire was used to estimate physical activity levels in RS-I and II and LASA Physical Activity questionnaire was used for RS-III

^c^In fasting conditions

^d^Diet beverages intake data was not available for RS-I, therefore intake data is only from RS-II and RS-III

^e^Alcoholic beverages intake was assessed as standard glasses of alcohol consumed per day, with one standard glass of alcohol defined as containing 10 g of ethanol

^f^Diet quality score assessed adherence to the Dutch Dietary Guidelines 2015 with a theoretical range from 0 to 14

### Healthy beverage index score and insulin resistance (HOMA-IR), risk of prediabetes and T2D

During 68,154 person-years of follow-up among 6052 participants (median follow-up 10 years), 1139 participants developed prediabetes (18.8%), and during 78,606 person-years of follow-up among 6718 participants (median follow-up 11 years), 784 participants developed T2D (11.7%). After multivariable adjustment (model 3), higher HBI score (per 10 points increment) was not associated with log-HOMA-IR levels (β: 0.003; 95% CI − 0.007, 0.014), nor with risk of prediabetes (HR: 1.01; 95% CI 0.97, 1.06), or T2D (HR: 1.01; 95% CI 0.96, 1.07) (Table 3). Similar results were obtained when stratifying the analyses by sub-cohort (Supplementary Tables 5 through 7). Associations did not differ by age, sex or BMI at baseline (all p-values for interaction > 0.05).

**Table 3:** Associations of total HBI score with insulin resistance (HOMA-IR), risk of prediabetes and T2D^a^

	HOMA-IR (n = 3896)		Prediabetes risk (n = 6052/1139 events)		T2D risk (n = 6718/784 events)	
	β	95% CI	HR	95% CI	HR	95% CI
Model 1^b^	0.004	(− 0.007, 0.017)	1.00	(0.96, 1.04)	1.01	(0.96, 1.07)
Model 2^c^	0.008	(− 0.004, 0.020)	1.02	(0.97, 1.06)	1.01	(0.96, 1.07)
Model 3^d^	0.003	(− 0.007, 0.014)	1.01	(0.97, 1.06)	1.01	(0.96, 1.07)

^a^Effect estimates are regression coefficients (β) for log HOMA-IR over time or hazard ratios (HR) for prediabetes or T2D risk with their corresponding 95% confidence intervals (95% CI) per 10 points increment in HBI score. Estimates are based on pooled results of three RS sub-cohorts

*HOMA-IR* homeostatic model assessment of insulin resistance, *RS* Rotterdam Study, *T2D* type 2 diabetes

^b^Adjusted for age, sex, total energy intake, RS sub-cohort, and time difference between HOMA-IR measurements (only for HOMA-IR analyses)

^c^Additionally adjusted for smoking status, educational level, physical activity, and diet quality score

^d^Additionally adjusted for body mass index

### Individual component scores and insulin resistance (HOMA-IR), risk of prediabetes and T2D

When examining the individual components of the HBI, we observed that after multivariable adjustment (model 3), meeting the criteria for getting the maximum score on coffee and tea (intake ≤ 40% of total fluid requirements, Table 1) and alcohol (intake ≤ 2 glasses/day for men and ≤ 1 glass/day for women) was associated with increased log-HOMA-IR (β: 0.044; 95% CI 0.009, 0.080; and β: 0.061; 95% CI 0.028, 0.094, respectively). In contrast, meeting the HBI criteria for getting the maximum score on low-fat milk (intake ≤ 16% of total fluid requirements) and SSBs (intake ≤ 8% of total fluid requirements) was associated with lower log-HOMA-IR (β: − 0.054; 95% CI − 0.091, − 0.016; and β: − 0.064; 95% CI − 0.127, − 0.002, respectively). Meeting the criteria for coffee and tea intake was also associated with higher risk of T2D (HR: 1.27; 95% CI 1.07, 1.49). No associations were found for meeting the criteria on any of the individual components and prediabetes risk (Table 4).

**Table 4:** Associations of individual component scores with insulin resistance (HOMA-IR), risk of prediabetes and T2D^a^

Components of the HBI	HOMA-IR (n = 3896)		Prediabetes risk (n = 6052/1139 events)		T2D risk (n = 6718/784 events)	
	β	95% CI	HR	95% CI	HR	95% CI
Water score 15 vs. < 15	− 0.001	(− 0.043, 0.033)	1.07	(0.92, 1.25)	1.01	(0.84, 1.21)
Coffee and tea score 5 vs. 0	0.044	(0.009, 0.080)	0.95	(0.83, 1.09)	1.27	(1.07, 1.49)
Low-fat milk score 5 vs. 0	− 0.054	(− 0.091, − 0.016)	0.93	(0.81, 1.07)	0.87	(0.74, 1.03)
Diet beverages score 5 vs. 0	− 0.038	(− 0.138, 0.062)	1.13	(0.65, 1.95)	0.80	(0.39, 1.67)
Juices score 5 vs. 0	0.009	(− 0.034, 0.054)	1.11	(0.92, 1.35)	1.16	(0.91, 1.47)
Alcohol score 5 vs. 0	0.061	(0.028, 0.094)	0.95	(0.83, 1.09)	1.05	(0.89, 1.24)
Full-fat milk score 5 vs. 0	− 0.006	(− 0.038, 0.024)	1.04	(0.89, 1.21)	1.04	(0.89, 1.21)
Sugar-sweetened beverage score 15 vs. 0	− 0.064	(− 0.127, − 0.002)	0.81	(0.66, 1.00)	0.90	(0.69, 1.18)
Energy from beverages score 20 vs. < 20	0.008	(− 0.027, 0.044)	0.96	(0.82, 1.12)	1.01	(0.84, 1.22)
Meeting total fluid requirements score 20 vs. < 20	0.006	(− 0.034, 0.047)	0.94	(0.81, 1.105)	0.94	(0.81, 1.10)

*HOMA-IR* homeostatic model assessment of insulin resistance, *RS* Rotterdam Study, *T2D* type 2 diabetes

^a^Effect estimates are regression coefficients (β) for log HOMA-IR over time or hazard ratios (HR) for prediabetes or T2D risk with their corresponding 95% confidence intervals (95% CI) per adherence vs. no-adherence or partial adherence to individual components criteria. Estimates are based on pooled results of three RS sub-cohorts using model 3. RS-1 was excluded from diet beverages score analyses due to lacking data on diet beverages consumption. Model 3 was adjusted for age, sex, total energy intake, RS sub-cohort, time difference between HOMA-IR measurements (only for HOMA-IR analyses), smoking status, educational level, physical activity, diet quality score, body mass index and all other component scores, except water analyses which were not adjusted for meeting total fluid requirements because of collinearity issues

### Sensitivity analyses

Alternative HBI scores 1 (leaving out scores from the components total energy from beverages and meeting total fluid requirements) and 2 (alternative score 1 additionally excluding scores from water), were also not associated with the study outcomes (Supplementary Table 8). The exclusion of each one of the 10 individual components from the total HBI score one by one at a time did not substantially change the estimates for all study outcomes, except for energy from beverages and prediabetes outcome. Excluding energy from beverages from the overall HBI was associated with higher risk of prediabetes (HR: 1.08; 95% CI 1.02, 1.15) (Supplementary Table 9). Also, no associations with any of the study outcomes were found when repeating main analyses using tertiles of the HBI score (higher vs. lower tertile) (Supplementary Table 10). Likewise, no significant associations were found when excluding participants with CVDs at baseline (Supplementary table 11). Finally, similar effect estimates were found when excluding participants that developed prediabetes and T2D within the first three years of follow-up compared to those obtained for the complete study populations for prediabetes and T2D analyses (Supplementary Table 12).

## Discussion

In summary, this study shows that overall beverage intake quality as reflected with total score in the Healthy Beverage Index (HBI) was not associated with insulin resistance (HOMA-IR) over time, nor with the incidence of prediabetes or T2D in our population of Dutch middle-aged and elderly people. Results from individual components showing differential associations with the study outcomes need to be interpreted with caution. Scores on individual components represent adherence to the components criteria and not actual beverage intake.

Our findings are partially in line with a previous study on the HBI and cardiometabolic health outcomes in US adults. A previous cross-sectional study that used data from the National Health and Nutrition Examination Survey (NHANES) 2005–2010 (n = 16,252 US adults), showed that higher HBI scores were associated with lower odds of high fasting insulin levels, but no association was observed with the odds of high fasting glucose levels [26]. Contrarily, in the current study we did not find any association between higher HBI scores and high fasting insulin levels in an exploratory analysis (Supplementary Table 13). Furthermore, we found no association between higher HBI scores and high glucose levels (Supplementary Table 13), nor with insulin resistance, risk of prediabetes, or T2D. In another European cohort the HBI was also computed, showing considerably higher scores compared to NHANES and our study (median: 89.7, IQR: 78.6–95). However, an association with cardiometabolic outcomes was also not found in that cohort [27]. Differences between the US and the current study might be partially explained by methodological differences such as in the study design. Our study, with a longitudinal design, had available data on repeated measurements over time of HOMA-IR as well as incidence of prediabetes and T2D, allowing us to make temporal inferences. Contrarily, the aforementioned study had a cross-sectional design and did not have data on repeated assessment of HOMA-IR over time or incident prediabetes or T2D. In addition, the use of different dietary assessment methods between the studies (average of two 24 h recalls vs. FFQs) might have led to differential misclassification of the exposure while assessing habitual beverage intake thought FFQs. However, mean ± SD HBI score was similar between the two populations (NHANES: 63 ± 13 vs. Rotterdam Study: 66.8 ± 14.4) [26].

Differences in findings between studies could also be explained by dissimilarities in beverage intake patterns, and how the HBI scoring system performs under different beverage intake patterns. Although the HBI was developed based on intake patterns and beverage recommendations for US adults, our study revealed contrasting findings in a population of Dutch middle-aged and elderly individuals. While SSBs consumption is relatively high among US adults [40], with energy from beverages accounting for up to 21% of their total energy intake [4], we observed that SSBs intake was comparatively  low in our population. Instead, coffee and tea intakes were relatively high, and energy from beverages constituted approximately 10% of their total energy intake.

Concerns on whether the HBI therefore adequately reflects a healthy beverage intake pattern in our study population arise. We found a low correlation between a diet quality index score that reflects adherence to the Dutch dietary guidelines 2015 and the HBI score, which reflects adherence to healthy beverage intake recommendations (*rho* = 0.21, p < 0.001), while previous studies suggest that healthy beverage patterns are associated with healthy dietary patterns [41]. This may indicate that Dutch and American dietary guidelines have different healthy beverage patterns. Finally, measurement error could also have been a cause of the differences between studies. Intake of some beverages like diet beverages in RS-I and water in all sub-cohorts may not have been appropriately estimated because of the nature of the FFQs, which may have affected total scores in the HBI.

Some of our findings for scores on individual components are not directly in line with evidence from previous observational studies on actual beverages intake (e.g., in mL or glasses per day). For example, a high coffee and tea intake that surpasses the maximum limit established for this component (> 40% of total fluid requirements) results in getting the lowest score (0 points). This likely explains why we found an association for higher score on the coffee and tea component (maximum score for intake ≤ 40% of total fluid requirements) with higher HOMA-IR and T2D risk, as a higher score actually corresponds to low consumption of coffee and tea. Coffee and tea were the main contributors to beverage intake in our study (median intake: 750, IQR: 550, 1000), and similar intakes have been reported in other European populations [42, 43]. In observational cohort studies, including the RS cohort [44], higher coffee and tea consumption was consistently associated with lower T2D risk [15, 16, 18]. The associations between the individual components coffee and tea, low-fat milk, alcohol and SSBs with the study outcomes need to be interpreted with caution. We found no  significant associations between the HBI score and the study outcomes when excluding individual components from the overall score (one at a time) in sensitivity analyses, in order to explore specific components as potential drivers of the associations. After exclusion of energy from beverages component, we noted a statistically significant association between the HBI score and prediabetes; however, such an association was not observed for HOMA-IR and T2D, and not in line with the main findings for prediabetes, suggesting that it is likely attributable to chance.

The HBI presents concerns for our study population. It was developed based on US intake patterns and may not reflect beverage quality accurately for populations with different intake distributions, like our Dutch participants. The scoring system's limited differentiation between healthier and less healthy beverage patterns poses challenges. Measuring total water intake is methodologically complex, impacting the accuracy of the HBI. The index places greater weight on certain  components, such as low SSBs intake, while neglecting the importance of other beverages like coffee and tea. Determining the significance of  beverage groups for general or cardiometabolic health is population-dependent. Previous research suggests that interventions targeting SSBs reduction and increased physical activity impact  component scores beyond SSBs intake [45]. In our study, Dutch participants adhered more to recommendations for diet drinks, SSBs, and juices, while US adults adhered to recommendations for coffee and tea, milk, fruit juice, alcohol, and meeting total fluid requirements Considering these concerns, the interpretation of HBI scores should account for diverse populations and contextual factors.

Results from sensitivity analyses confirmed our main findings and showed no associations for alternative HBI scores with the study outcomes. Also, assessing HBI scores in tertiles did not change the main results.

Our study has several strengths. First, to our knowledge, this is the first study investigating longitudinal associations between overall beverage intake quality and our study outcomes for which we had available data of a large European population with repeated measurements of HOMA-IR and a long follow-up for prediabetes and T2D risk (> 10 years). Second, we added to scarce evidence on overall beverage intake quality and cardiometabolic health specifically T2D risk, by assessing associations with early risk stages of the disease, namely HOMA-IR and prediabetes risk in a longitudinal study, which in turn may help to understand how overall beverage intake quality is associated with T2D development. Third, the use of plasma glucose concentrations to assess prediabetes and T2D is another strength of our study. A high proportion of individuals with T2D is undiagnosed, and these cases would have not been detected by self-report. This is relevant for both, baseline (individuals with undiagnosed diabetes would not have been excluded from the analyses) and case ascertainment. Last, we performed a series of additional and sensitivity analyses to comprehensively examine associations between different HBI components and alternative versions of the HBI with the study outcomes, with the purpose of evaluating the HBI.

This study also had some limitations. First, measurement errors were likely to occur, as the intake of some beverages such as diet beverages in RS-I and water in all sub-cohorts may not have been appropriately estimated because of the nature of the FFQs. Although this might have affected the total HBI score, its average was similar to that of US adults in a previous study, and water intake in the Dutch population is generally low (median: 400 g/day) [46]. Second, we used two different FFQs with different numbers of items, however, the FFQs are equally capable of estimating overall beverage intake quality, since sensitivity analyses showed no major differences in beverage intake quality and in the estimates between sub-cohorts with different follow-up periods. Third, although we adjusted for many possible confounders, we cannot completely rule out residual confounding. Finally, our results cannot be generalized to other populations with different age, race and beverage intake patterns, and need further replication in other cohorts.

In conclusion, our findings within a large population-based cohort suggest that overall beverage intake quality as assessed with the healthy beverage index is not associated with insulin resistance (HOMA-IR) over time, nor with prediabetes or T2D risk in middle-age and elderly Dutch. Results of individual HBI components need to be interpreted with caution because of the complex nature of the HBI, which may not reflect true associations between actual beverage intake and disease outcomes. The HBI may not be an optimal scoring system for this population, FFQ data and/or these outcomes. Other means to evaluate overall beverage intake quality should be explored. Future studies should focus on developing country-specific scoring systems that appropriately assess overall beverage intake, and test how changes in individual components affect other components (replacement) and therefore scores and overall quality of beverage intake.

## Supplementary Information

Below is the link to the electronic supplementary material.

Supplementary file1 (DOCX 176 kb)

## Data Availability

Data described in the manuscript, code book, and analytic code will be made available upon request pending application and approval.

## Abbreviations

Β: Beta coefficient
BMI: Body mass index
CI: Confidence interval
FFQs: Food-frequency questionnaires
HBI: Healthy beverage index
HOMA-IR: Homeostatic model assessment of insulin resistance
HR: Hazard ratio
IQR: Interquartile range
LMM: Linear mixed models
MET: Metabolic equivalent of task
RS: Rotterdam Study
SD: Standard deviation
SSBs: Sugar-sweteened beverages
T2D: Type 2 diabetes
WHO: World Health Organization
