# Supplementary material for: The healthy beverage index is not associated with insulin resistance, prediabetes and type 2 diabetes risk in the Rotterdam Study
Source: Eur J Nutr. 2023 Jul 25;62(7):3021–31. doi: 10.1007/s00394-023-03209-6 (PMC10468439; doi:10.1007/s00394-023-03209-6)
Supplement: Supplementary file 1 — Supplementary file1 (DOCX 176 kb) [file 394_2023_3209_MOESM1_ESM.docx]

**SUPPLEMENTARY MATERIAL:**

**The Healthy Beverage Index is not associated with insulin resistance, prediabetes and type 2 diabetes risk in the Rotterdam Study**

Maria G. Jacobo Cejudo^1,2^*, Carolina Ochoa-Rosales^2,3^, [Fariba Ahmadizar](https://pubmed.ncbi.nlm.nih.gov/?term=Ahmadizar+F&cauthor_id=32746929)^2,4^, Maryam Kavousi^2^, Johanna M. Geleijnse^1^, Trudy Voortman^1,2^

^1^Division of Human Nutrition and Health, Wageningen University, PO Box 17, 6700, AA, Wageningen, The Netherlands; [marianne.geleijnse@wur.nl](mailto:marianne.geleijnse@wur.nl) (JMG).

^2^Department of Epidemiology, Erasmus MC, University Medical Center Rotterdam, The Netherlands; [f.ahmadizar@erasmusmc.nl](mailto:f.ahmadizar@erasmusmc.nl) (FA); [m.kavousi@erasmusmc.nl](mailto:m.kavousi@erasmusmc.nl) (MK); [trudy.voortman@erasmusmc.nl](mailto:trudy.voortman@erasmusmc.nl) (TV).

^3^Latin American Brain Health Institute (BrainLat), Universidad Adolfo Ibáñez, Santiago, Chile; [carolina.ochoa@uai.cl](mailto:carolina.ochoa@uai.cl)

^4^Julius Center for Health Sciences and Primary Care, University Medical Center Utrecht.

*Correspondence: [m.jacobocejudo@erasmusmc.nl](mailto:m.jacobocejudo@erasmusmc.nl) (MGJC); Tel.: +31-629-155870

**Supplementary Figure 1.** Flow-chart of participants selection.

Participants at baseline:

n=14,926

(RS-I: 7,983; RS-II: 3,011, RS-III: 3,932)

Participants additionally excluded for analyses of insulin resistance (HOMA-IR): n= 2,873

Reasons for exclusion:

- Having less than 2 measurements of HOMA-IR

Participants additionally excluded: n= 2,931

Reasons for exclusion:

- Prevalent type 2 diabetes (T2D) at baseline: n=963
- Missing data on prevalent T2D at baseline: n=1,968

Participants free of T2D at baseline

n=6,769

(RS-I: 2,971; RS-II: 1,412; RS-III: 2,386)

Participants additionally excluded for analyses of prediabetes: n= 717

Reasons for exclusion:

- Prediabetes at baseline
- Missing prediabetes follow-up data

Participants for analyses of T2D

n=6,718

(RS-I: 2,935; RS-II: 1,400; RS-III: 2,383)

Participants additionally excluded for analyses of T2D: n= 51

Reasons for exclusion:

- Missing T2D follow-up data

Participants for analyses of HOMA-IR

n=3,896

(RS-I: 1,172; RS-II: 893; RS-III: 1,831)

Participants for analyses of prediabetes

n=6,052

(RS-I: 2,617; RS-II: 1,249; RS-III: 2,186)

Participants excluded: n= 5,226

Reasons for exclusion:

- Missing data on dietary intake: n=5,176
- Implausibly energy intake: n=50

Participants with valid dietary data at baseline

n=9,700

(RS-I: 5,433; RS-II: 1,623; RS-III: 2,644)

| **Supplementary Table 1.** Beverage items included in the components of the HBI by sub-cohort^a^. | | | |
| --- | --- | --- | --- |
| **Beverage components** | **RS-I** | **RS-II** | **RS-III** |
| Water | -Tap water  -Mineral water | -Tap water  -Mineral water | -Mineral water |
| Coffee and tea | -Coffee ready to drink  -Black tea | -Coffee ready to drink  -Caffeine free coffee  -Green tea  -Black tea | -Coffee ready to drink  -Green tea  -Black tea |
| Low-fat milk (fat content < 2.0%) | -Skimmed-milk  -Semi-skimmed milk  -Buttermilk  -Skimmed chocolate milk | -Skimmed-milk  -Semi-skimmed milk  -Buttermilk  -Skimmed chocolate milk  -Semi-skimmed chocolate milk | -Skimmed-milk  -Semi-skimmed milk  -Buttermilk  -Skimmed chocolate milk  -Semi-skimmed chocolate milk  - Diet coffee milk |
| Diet beverages | N.A. | -Cola light  -Other diet sodas | -Diet sodas |
| Fruit and vegetable juices | -Apple juice  -Orange juice  -Grapefruit juice  -Fresh lemon juice  -Tomato juice  -Tomato and other  vegetable juices | -Orange juice  -Grapefruit juice  -Other fruit juices  -Fruit nectar  -Tomato juice | -Orange juice  -Other fruit juices  -Vegetable juice |
| Alcoholic beverages | -Beer  -Red wine  -White wine  -Gin  -Sherry | -Beer  -Red wine  -White wine  -Gin  -Sherry  -Advocaat  -Strong liquor  -Liquor  -Campari  - Martini  -Port  -Vermouth | -Beer  -Red wine  -White wine  -Advocaat  - Strong liquor |
| Full-fat milk (fat content ≥ 2.0 %) | -Raw milk  -Whole milk  -Whole chocolate milk  -Coffee creamer with added PUFAs  -Whole coffee creamer  -Semi-skimmed coffee creamer | -Whole milk  -Whole chocolate milk  -Regular coffee creamer | -Whole milk  -Whole chocolate milk  -Whole coffee creamer  -Semi-skimmed coffee creamer |
| Sugar-Sweetened beverages | -Cola soft drink with caffeine  -Soft drink without caffeine  -Alcohol free beer  -Fruit drink | -Cola soft drink with caffeine  -Soft drink without caffeine  -Alcohol free beer | -Soft drink without caffeine  -Alcohol free beer  -Breakfast drink |
| Energy from beverages | -All beverages | -All beverages | -All beverages |
| Total fluid requirements | -All beverages | -All beverages | -All beverages |

^a^Diet beverages intake was not assessed in RS-I. Soy beverages were not included in low-fat and full-fat milk components because of their different nutritional composition, and because in the Netherlands they are not fortified. Herbal infusions were also not included in coffee and tea component. However, soy beverages and herbal teas were taken into account for the calculation of the scores for the components energy from beverages and meeting total fluid requirements. In consumers, overall median (IQR) intakes of herbal teas and soy beverages were 150 (67-281) mL/day and 96 (21-182) mL/day, respectively.

**Supplementary Table 2.** Distribution of individual component scores across tertiles of total HBI score in 6,769 participants of the Rotterdam study^a^.

|  |  | Tertiles of the HBI score | | |
| --- | --- | --- | --- | --- |
|  | Overall  n=6,769 | T1  n=2,254 | T2  n=2,254 | T3  n=2,261 |
| Water score | 6.5 (0-15) | 0.4 (0-6) | 4 (0-15) | 15 (9-15) |
| Coffee and tea score | 5.0 (0-5) | 5 (0-5) | 5 (0-5) | 5 (0-5) |
| Low-fat milk score | 5.0 (5-5) | 5 (0-5) | 5 (5-5) | 5 (5-5) |
| Diet beverages score | 5.0 (5-5) | 5 (5-5) | 5 (5-5) | 5 (5-5) |
| Fruit and vegetable juices score | 5.0 (5-5) | 5 (5-5) | 5 (5-5) | 5 (5-5) |
| Alcohol score | 5.0 (0-5) | 0 (0-5) | 5 (0-5) | 5 (5-5) |
| Full-fat milk score | 5.0 (0-5) | 0 (0-5) | 5 (0-5) | 5 (0-5) |
| Sugar-sweetened beverages score | 15.0 (15-15) | 15 (15-15) | 15 (15-15) | 15 (15-15) |
| Total beverage energy score | 12.7 (0-20) | 0 (0-4) | 15 (1-20) | 20 (20-20) |
| Meeting total fluid requirements score | 14.8 (11-20) | 14.3 (11-18) | 13 (10-19) | 17 (13-20) |
| Total HBI score | 66.8 ± 14.4 | 50.7 ± 7.4 | 67.0 ± 3.4 | 82.7 ± 6.8 |

^a^Total HBI score ranges from 0 to 100. Individual component scores are shown as median (interquartile range) because of their skewed distribution. Total HBI score is shown as mean ± SD because of its normal distribution

**
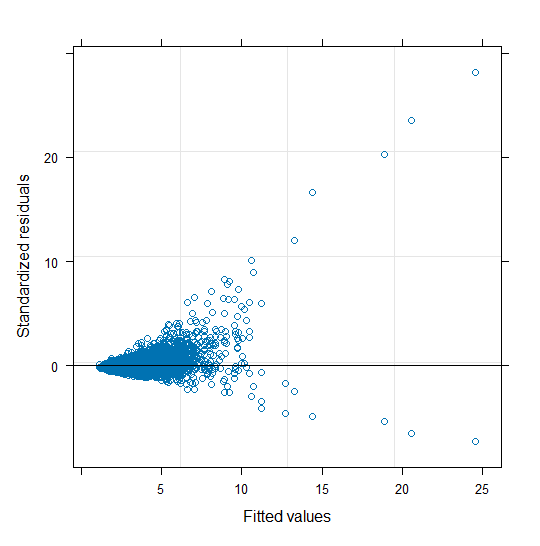
Supplementary Figures 2 and 3.** Residuals plots with histograms.

**
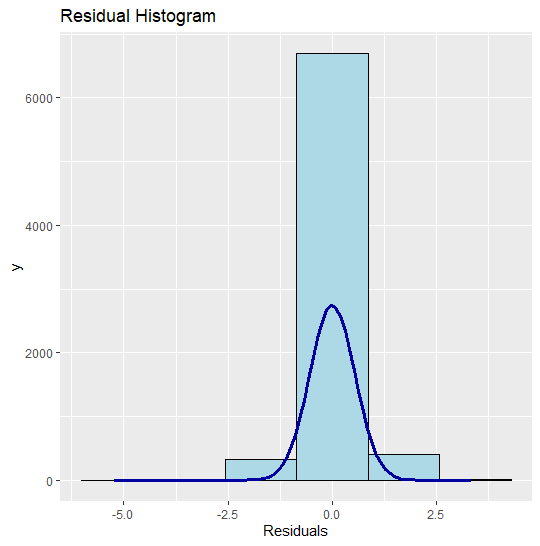

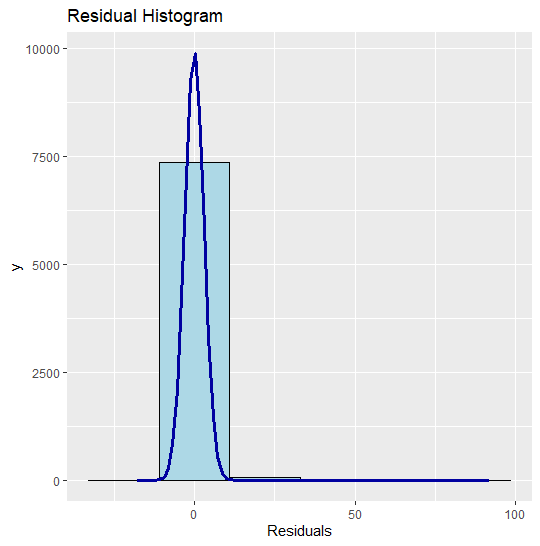
**
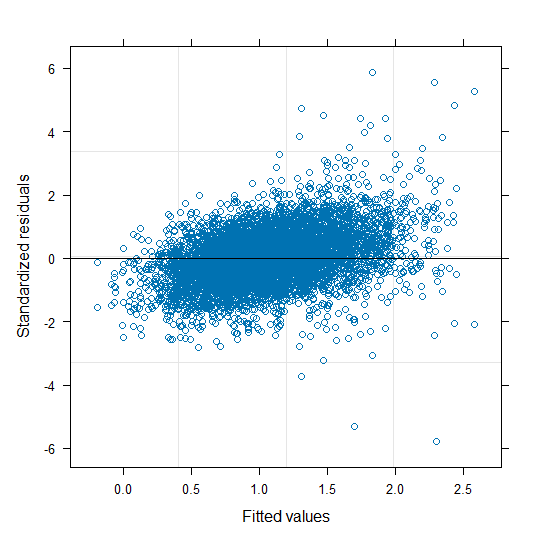


**Figure 2.** Residuals plot and histogram before log-transformation of HOMA-IR values

**Figure 3.** Residuals plot and histogram after log-transformation of HOMA-IR values

**Supplementary Figure 4.** Natural cubic spline of the association between HBI score (per 10 points increment) and prediabetes risk^a^.


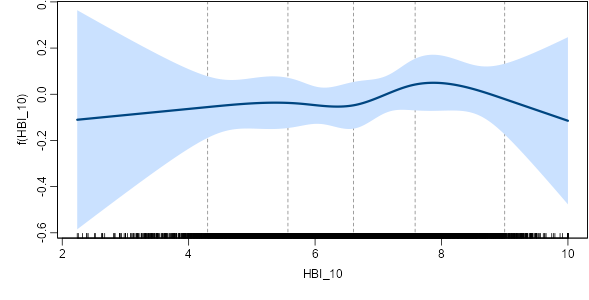


^a^Non-linearity was tested using most adjusted model (model 3). Model 3 was adjusted for age, sex, total energy intake, RS sub-cohort, smoking status, educational level, physical activity, diet quality score, and body mass index. Abbreviations: Rotterdam Study. P nonlinearity = 0.82.

**Supplementary Figure 5.** Natural cubic spline of the association between HBI score (per 10 points increment) and T2D risk^a^.

**
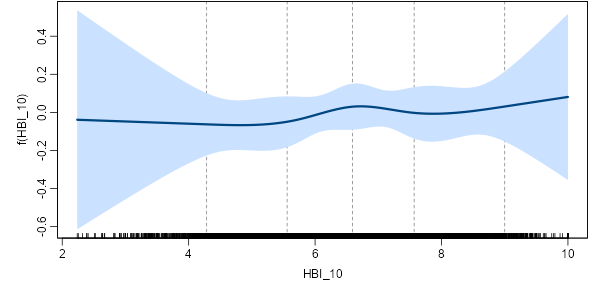
**

^a^Non-linearity was tested using most adjusted model (model 3). Model 3 was adjusted for age, sex, total energy intake, RS sub-cohort, smoking status, educational level, physical activity, diet quality score, and body mass index. Abbreviations: Rotterdam Study; T2D, type 2 diabetes. P nonlinearity = 0.93.

**Supplementary Table 3.** Baseline characteristics of the study participants by sub-cohort^a^.

|  | RS-I  n= 2,971 | RS-II  n=1,412 | RS-III  n=2,386 |
| --- | --- | --- | --- |
| Age (years) | 65.5 ± 6.7 | 63.6 ± 7.2 | 56.8 ± 6.4 |
| Sex, n (% female) | 1,770 (60%) | 778 (55%) | 1,429 (60%) |
| Smoking, n (%) |  |  |  |
| Never | 1,020 (34%) | 416 (30%) | 769 (32%) |
| Former | 1,318 (44%) | 715 (51%) | 1,113 (47%) |
| Current | 633 (21%) | 281 (20%) | 504 (21) |
| Educational level, n (%)  Primary  Lower  Intermediate  Higher | 467 (16%)  1,293 (44%)  895 (30%)  299 (10%) | 100 (7%)  639 (46%)  402 (29)  252 (18) | 227 (10%)  820 (34%)  650 (27%)  682 (29%) |
| Physical activity (METh/w)^b^ | 80.7 (55.4, 116.3) | 77.3 (52.9, 104.7) | 42.9 (17.7, 82.5) |
| BMI, (kg/m^2^) | 26.3 ± 5.5 | 27.0 ± 4.0 | 27.2 ± 4.3 |
| Plasma glucose (mmol/L)^c^ | 5.50 (5.10, 5.90) | 5.50 (5.20, 5.90) | 5.20 (4.90, 5.60) |
| Serum insulin (pmol/L)^c^ | 64.0 (46.0, 91.0) | 68.0 (50.0, 94.0) | 75.0 (54.0, 107.0) |
| HOMA-IR^2^ | 3.20 ± 2.62 | 3.40 ± 2.91 | 3.60 ± 3.00 |
| Dietary intake |  |  |  |
| Total energy (kcal/day) | 1,983 ± 502 | 2,204 ± 569 | 2,310 ± 714 |
| Energy from beverages (kcal/day) | 215 (137, 317) | 279 (182, 395) | 228 (135, 350) |
| Water, n(%) of consumers  mL/day only in consumers  Coffee and tea, n(%) of consumers  mL/day only in consumers | 2,298 (77%)  349 (175, 524)  2,962 (99.7%)  875 (678, 1,000) | 1,013 (72%)  524 (349, 960)  1,399 (99.1%)  800 (625, 1,075) | 1,413 (59%)  268 (96, 557)  2,346 (98.3%)  580 (406, 812) |
| Low-fat milk, n(%) of consumers  mL/day only in consumers  Diet beverages, n(%) of consumers  mL/day only in consumers  Juices, n(%) of consumers  mL/day only in consumers  Alcohol, n(%) of consumers  glasses/day only in consumers^d^  Full-fat milk, n(%) of consumers  mL/day only in consumers  SSBs, n(%) of consumers  mL/day only in consumers  Total beverage (mL/day)  Total HBI score  Diet quality score^e^ | 2,274 (77%)  232 (165, 404)  N.A.  N.A.  1,374 (46%)  40 (17, 100)  2,479 (83%)  0.74 (0.15, 2.00)  1,473 (50%)  40 (24.0, 120.0)  1,347 (45%)  50.0 (24.0, 150.0)  1,577 (1,289, 1,945) | 1.027 (73%)  230 (165, 449)  217 (15%)  87.3 (37, 175.0)  1,071 (76%)  140 (49, 256)  1,203 (85%)  1.10 (0.32, 2.50)  531 (38%)  32.0 (24.0, 48.0)  341 (24%)  74.7 (25.0, 175.0)  1,884 (1,463, 2,366) | 1,951 (82%)  153 (96, 284)  688 (29%)  54.0 (21.4, 161.0)  1,826 (77%)  80 (27, 139)  1,994 (84%)  1.20 (0.41, 2.30)  783 (33%)  53.6 (13.0, 139.3)  904 (38%)  44.0 (21.0, 109.3)  1,329 (1,032, 1,688) |
|  | 65.2 ± 14.6 | 69.3 ± 14.4 | 67.4 ± 13.9 |
|  | 6.9 ± 1.8 | 6.1 ± 1.8 | 7.0 ± 1.9 |

^a^Values are mean ± SD for continuous variables with normal distribution, median (IQR) for continuous variables with a skewed distribution or absolute numbers and percentages for categorical variables, based on non-imputed data. ^b^Zutphen Physical Activity questionnaire was used to estimate physical activity levels in RS-I and II and LASA Physical Activity questionnaire was used for RS-III. ^c^ In fasting conditions. ^d^Alcoholic beverages intake is presented as standard glasses of alcohol per day and 1 standard glass of alcohol was considered as 10 grams of alcohol. ^e^ Diet quality score assessed adherence to Dutch Dietary Guidelines 2015 with a theoretical range from 0 to 14. Missing data for variables was as follows:n=43 for educational level, n=265 for physical activity, n= 87 for BMI, n=151 for blood glucose, n=307 for serum insulin, n=331 for HOMA-IR, and n=2,971 for ASBs (all RS-I). Abbreviations: RS, Rotterdam Study; SD, standard deviation; IQR, interquartile range; HOMA-IR, Homeostatic model assessment of insulin resistance; N.A, not-applicable; SSBs, sugar-sweetened beverages; HBI, healthy beverage index score.

**Supplementary Table 4.** Participant’s percentage of adherence to the HBI components^a^.

|  | Overall  n=6,769 | RS-I  n=2,971 | RS-II  n=1,412 | RS-III  n=2,386 |
| --- | --- | --- | --- | --- |
| Total water intake ≥20% of total fluid requirements | 31% | 35% | 41% | 18% |
| Coffee and tea ≤40% of total fluid requirements | 58% | 41% | 56% | 81% |
| Low-fat milk ≤16% of total fluid requirements | 77% | 70% | 75% | 85% |
| Diet drinks ≤16% of total fluid requirements | 98% | N.A. | 98% | 98% |
| Fruit and vegetable juices ≤8% of total fluid requirements | 87% | 94% | 71% | 88% |
| Alcohol ≤1 glass/day for women or ≤2 glasses/day for men | 67% | 71% | 64% | 64% |
| Full-fat milk 0% of total fluid requirements | 59% | 50% | 62% | 67% |
| Sugar-sweetened beverages ≤8% of total fluid requirements | 93% | 91% | 94% | 94% |
| Total energy from beverages ≤10% of total energy intake | 42% | 42% | 32% | 49% |
| Total beverage intake (mL/d) ≥ total fluid requirements ^a^ | 23% | 29% | 36% | 9% |

^a^Values represent the percentage of the population that adhered to the recommended intakes of beverage (groups), energy from beverages and total beverages. Total fluid requirements = 1 ml of fluid consumed per 1 kcal per day.

**Supplementary Table 5.** Associations of the HBI score with insulin resistance (HOMA-IR) by Rotterdam Study (RS) sub-cohort^a^.

| **HOMA-**  **IR** | RS-I (n=1,172) | |  | RS-II (n=893) | |  | RS-III (n=1,831) | |
| --- | --- | --- | --- | --- | --- | --- | --- | --- |
|  | β | 95% CI |  | β | 95% CI |  | β | 95% CI |
| Model 1^b^ | -0.001 | (-0.023, 0.019) |  | 0.002 | (-0.022, 0.028) |  | 0.009 | (-0.008, 0.028) |
| Model 2^c^ | 0.0001 | (-0.021, 0.022) |  | 0.002 | (-0.023, 0.028) |  | 0.017 | (-0.0007 0.036) |
| Model 3^d^ | 0.002 | (-0.018, 0.023) |  | -0.004 | (-0.026, 0.017) |  | 0.003 | (-0.012, 0.020) |

^a^Effect estimates are regression coefficients (β) with 95% confidence intervals (95% CI) for log-HOMA-IR over time per 10 points increment in HBI score. ^b^Adjusted for age, sex, total energy intake, and time difference between HOMA-IR measurements. ^c^Additionally adjusted for smoking status, educational level, physical activity, and diet quality score. ^d^Additionally adjusted for body mass index. Abbreviations: HOMA-IR, homeostatic model assessment of insulin resistance.

**Supplementary Table 6.** Associations of the HBI score with prediabetes risk by Rotterdam Study (RS) sub-cohort^a^.

| **Prediabetes** | RS-I (n=2,617/519) | |  | RS-II (n=1,249/290) | |  | RS-III (n=2,186/330) | |
| --- | --- | --- | --- | --- | --- | --- | --- | --- |
|  | HR | 95% CI |  | HR | 95% CI |  | HR | 95% CI |
| Model 1^b^ | 1.01 | (0.94, 1.07) |  | 0.97 | (0.90, 1.06) |  | 1.01 | (0.92, 1.09) |
| Model 2^c^ | 1.01 | (0.95, 1.08) |  | 1.00 | (0.92, 1.08) |  | 1.05 | (0.96, 1.14) |
| Model 3^d^ | 1.01 | (0.95, 1.08) |  | 0.98 | (0.90, 1.07) |  | 1.03 | (0.95, 1.12) |

^a^Effect estimates are hazard ratios (HR) with 95% confidence intervals (95% CI) for prediabetes risk per 10 points increment in HBI score. ^b^Adjusted for age, sex, and total energy intake. ^c^Additionally adjusted for smoking status, educational level, physical activity, and diet quality score. ^d^Additionally adjusted for body mass index.

**Supplementary Table 7.** Associations of the HBI score with T2D risk by Rotterdam Study (RS) sub-cohort^a^.

| **T2D** | RS-I (n=2,935/434) | |  | RS-II (n=1,400/195) | |  | RS-III (n=2,383/155) | |
| --- | --- | --- | --- | --- | --- | --- | --- | --- |
|  | HR | 95% CI |  | HR | 95% CI |  | HR | 95% CI |
| Model 1^b^ | 0.99 | (0.93, 1.06) |  | 1.09 | (0.98, 1.21) |  | 0.99 | (0.88, 1.13) |
| Model 2^c^ | 0.98 | (0.92, 1.05) |  | 1.10 | (0.98, 1.23) |  | 1.00 | (0.88, 1.14) |
| Model 3^d^ | 0.99 | (0.92, 1.05) |  | 1.09 | (0.97, 1.21) |  | 1.00 | (0.88, 1.14) |

^a^Effect estimates are hazard ratios (HR) with 95% confidence intervals (95% CI) for T2D risk per 10 points increment in HBI score. ^b^Adjusted for age, sex, and total energy intake. ^c^Additionally adjusted for smoking status, educational level, physical activity, and diet quality score. ^d^Additionally adjusted for body mass index. Abbreviations: T2D, type 2 diabetes.

**Supplementary Table 8.** Associations of alternative HBI scores 1 and 2 with insulin resistance (HOMA-IR), risk of prediabetes and T2D (Sensitivity analyses)^a^.

|  |  |  |  |  |  |  |  |  |  | Alternative HBI scores |  | HOMA-IR  (n=3,896) | | Prediabetes risk  (n=6,052/1,139 events) | | | | T2D risk  (n=6,718/784 events) | | |  |
| --- | --- | --- | --- | --- | --- | --- | --- | --- | --- | --- | --- | --- | --- | --- | --- | --- | --- | --- | --- | --- | --- |
|  |  |  |  |  |  |  |  |  |  |  |  | β | 95% CI |  | HR | 95% CI |  | HR | 95% CI |  |  |
|  |  |  |  |  |  |  |  |  |  | *Alternative score 1* |  |  |  |  |  |  |  |  |  |  |  |
|  |  |  |  |  |  |  |  |  |  | Model 1^b^ |  | 0.015 | (-0.004, 0.035) |  | 1.04 | (0.97, 1.11) |  | 1.06 | (0.97, 1.15) |  |  |
|  |  |  |  |  |  |  |  |  |  | Model 2^c^ |  | 0.019 | (-0.002, 0.041) |  | 1.07 | (0.99, 1.15) |  | 1.09 | (0.99, 1.20) |  |  |
|  |  |  |  |  |  |  |  |  |  | Model 3^d^ |  | 0.007 | (-0.012, 0.027) |  | 1.06 | (0.98, 1.14) |  | 1.09 | (0.99, 1.20) |  |  |
|  |  |  |  |  |  |  |  |  |  | *Alternative score 2* |  |  |  |  |  |  |  |  |  |  |  |
|  |  |  |  |  |  |  |  |  |  | Model 1^b^ |  | -0.005 | (-0.035, 0.024) |  | 0.92 | (0.84, 1.02) |  | 1.05 | (0.93, 1.20) |  |  |
|  |  |  |  |  |  |  |  |  |  | Model 2^c^ |  | 0.001 | (-0.033, 0.036) |  | 0.98 | (0.87, 1.10) |  | 1.02 | (0.88, 1.19) |  |  |
|  |  |  |  |  |  |  |  |  |  | Model 3^d^ |  | -0.015 | (-0.047, 0.015) |  | 0.95 | (0.84, 1.07) |  | 1.00 | (0.86, 1.16) |  |  |

^a^Effect estimates are regression coefficients (β) for log- HOMA-IR over time or hazard ratios (HR) for prediabetes or T2D risk with their corresponding 95% confidence intervals (95% CI) per 10 points increment in alternative scores 1 and 2. Alternative score 1 is total HBI score excluding scores from energy from beverages and meeting total fluid requirements components. Alternative score 2 is alternative score 1 additionally excluding scores from water component. Estimates are based on pooled results of three RS sub-cohorts. ^b^Adjusted for age, sex, total energy intake, RS sub-cohort, and time difference between HOMA-IR measurements (only for HOMA-IR analyses). ^c^Additionally adjusted for smoking status, educational level, physical activity, diet quality score and the excluded components. ^d^Additionally adjusted for body mass index. Abbreviations: HOMA-IR, homeostatic model assessment of insulin resistance; Rotterdam Study; T2D, type 2 diabetes.

**Supplementary Table 9.** Associations of the HBI score with insulin resistance (HOMA-IR), risk of prediabetes and T2D after excluding each one of 10 components one by one, and additionally adjusting for the excluded one (Sensitivity analyses)^a^.

|  | HBI with 9 components instead of 10 | | | | | | | | | |  | HOMA-IR  (n=3,896) | | Prediabetes risk  (n=6,052/1,139 events) | | | | T2D risk  (n=6,718/784 events) | |  |
| --- | --- | --- | --- | --- | --- | --- | --- | --- | --- | --- | --- | --- | --- | --- | --- | --- | --- | --- | --- | --- |
|  |  |  |  |  |  |  |  |  |  |  |  | β | 95% CI |  | HR | 95% CI |  | HR | 95% CI |  |
|  |  |  |  |  |  |  |  |  |  | Excluding water |  | -0.0007 | (-0.014, 0.013) |  | 0.96 | (0.91, 1.01) |  | 0.99 | (0.92, 1.06) |  |
|  |  |  |  |  |  |  |  |  |  | Excluding coffee and tea |  | 0.004 | (-0.006, 0.015) |  | 1.03 | (0.99, 1.07) |  | 1.03 | (0.98, 1.08) |  |
|  |  |  |  |  |  |  |  |  |  | Excluding low-fat milk |  | 0.007 | (-0.004, 0.020) |  | 1.03 | (0.98, 1.08) |  | 1.02 | (0.96, 1.08) |  |
|  |  |  |  |  |  |  |  |  |  | Excluding diet beverages |  | 0.003 | (-0.007, 0.014) |  | 1.01 | (0.97, 1.06) |  | 1.01 | (0.96, 1.07) |  |
|  |  |  |  |  |  |  |  |  |  | Excluding fruit and vegetable juices |  | 0.001 | (-0.009, 0.013) |  | 1.01 | (0.96, 1.05) |  | 1.00 | (0.95, 1.06) |  |
|  |  |  |  |  |  |  |  |  |  | Excluding alcohol |  | -0.007 | (-0.019, 0.005) |  | 1.03 | (0.98, 1.08) |  | 1.00 | (0.94, 1.06) |  |
|  |  |  |  |  |  |  |  |  |  | Excluding full-fat milk |  | 0.006 | (-0.004, 0.018) |  | 1.00 | (0.96, 1.05) |  | 1.02 | (0.97, 1.08) |  |
|  |  |  |  |  |  |  |  |  |  | Excluding sugar-sweetened beverages |  | 0.009 | (-0.002, 0.021) |  | 1.04 | (0.99, 1.09) |  | 1.03 | (0.97, 1.09) |  |
|  |  |  |  |  |  |  |  |  |  | Excluding total beverage energy |  | 0.002 | (-0.014, 0.018) |  | 1.08 | (1.02, 1.15) |  | 1.03 | (0.95, 1.11) |  |
|  |  |  |  |  |  |  |  |  |  | Excluding meeting total fluid requirements |  | 0.003 | (-0.007, 0.015) |  | 1.01 | (0.96, 1.05) |  | 1.01 | (0.96-1.07) |  |

^a^Effect estimates are regression coefficients (β) for log HOMA-IR over time or hazard ratios (HR) for prediabetes or T2D risk with their corresponding 95% confidence intervals (95% CI) per 10 points increment in HBI score by excluding one by one of 10 individual components at a time and additionally adjusting for the excluded component. Estimates are based on pooled results of three RS sub-cohorts using model 3. Model 3 was adjusted for age, sex, total energy intake, RS sub-cohort, time difference between HOMA-IR measurements (only for HOMA-IR), smoking status, educational level, physical activity, diet quality score, body mass index and the excluded component. RS-1 was excluded from analyses of diet beverages due to lack of diet beverages intake data in that sub-cohort. Abbreviations: HOMA-IR, homeostatic model assessment of insulin resistance; Rotterdam Study; T2D, type 2 diabetes.

**Supplementary Table 10.** Associations of tertiles of the HBI score with insulin resistance (HOMA-IR), risk of prediabetes and T2D (Sensitivity analyses)^a^.

|  | n / events | T1  < 60.70 | T2  ≥60.70 - < 72.82 | T3  ≥72.82 |
| --- | --- | --- | --- | --- |
| *HOMA-IR* | n=3,896 |  |  |  |
| Model 1^b^ |  | Reference | -0.028 (-0.069, 0.012 ) | 0.024 (-0.017, 0.066) |
| Model 2^c^ |  | Reference | -0.020 (-0.061, 0.020 ) | 0.036 (-0.005, 0.078) |
| Model 3^d^ |  | Reference | -0.012 (-0.049, 0.024) | 0.019 (-0.019, 0.057) |
| *Prediabetes* | n=6,052/ 1,139 events |  |  |  |
| Model 1^b^ |  | Reference | 1.02 (0.88, 1.18) | 1.04 (0.90, 1.21) |
| Model 2^c^ |  | Reference | 1.06 (0.92, 1.23) | 1.11 (0.95, 1.29) |
| Model 3^d^ |  | Reference | 1.07 (0.92, 1.24) | 1.10 (0.94, 1.28) |
| *T2D* | n=6,718/ 784 events |  |  |  |
| Model 1^b^ |  | Reference | 1.03 (0.86, 1.22) | 1.05 (0.88, 1.26) |
| Model 2^c^ |  | Reference | 1.04 (0.87, 1.25) | 1.05 (0.87, 1.26) |
| Model 3^d^ |  | Reference | 1.04 (0.87, 1.24) | 1.04 (0.86, 1.26) |

^a^Effect estimates are regression coefficients (β) for log HOMA-IR over time or hazard ratios (HR) for prediabetes or T2D risk with their corresponding 95% confidence intervals (95% CI) across tertiles of HBI score. Estimates are based on pooled results of three RS sub-cohorts. ^b^Adjusted for age, sex, total energy intake, RS sub-cohort, and time difference between HOMA-IR measurements (only for HOMA-IR analyses). ^c^Additionally adjusted for smoking status, educational level, physical activity, and diet quality score. ^d^Additionally adjusted for body mass index. Abbreviations: HOMA-IR, homeostatic model assessment of insulin resistance; Rotterdam Study; T2D, type 2 diabetes.

**Supplementary Table 11.** Associations of the HBI score with insulin resistance (HOMA-IR), risk of prediabetes and T2D after excluding participants with CVD at baseline (Sensitivity analyses)^a^.

|  | HOMA-IR  n=3,735 | | Prediabetes risk  n=5,696 / 1,077 events | | T2D risk  n=6,304 / 724 events | |
| --- | --- | --- | --- | --- | --- | --- |
|  | β | 95% CI | HR | 95% CI | HR | 95% CI |
| Model 1^b^ | 0.004 | (-0.008, 0.016) | 1.00 | (0.96, 1.04) | 1.01 | (0.96, 1.07) |
| Model 2^c^ | 0.007 | (-0.004, 0.020) | 1.02 | (0.97, 1.07) | 1.01 | (0.96, 1.07) |
| Model 3^d^ | 0.002 | (-0.008, 0.014) | 1.02 | (0.97, 1.06) | 1.01 | (0.95, 1.07) |

^a^Effect estimates are regression coefficients (β) for log HOMA-IR over time or hazard ratios (HR) for prediabetes or T2D risk with their corresponding 95% confidence intervals (95% CI) per 10 points increment in HBI score. Estimates are based on pooled results of three RS sub-cohorts. ^b^Adjusted for age, sex, total energy intake, RS sub-cohort, and time difference between HOMA-IR measurements (only for HOMA-IR analyses). ^c^Additionally adjusted for smoking status, educational level, physical activity, and diet quality score. ^d^Additionally adjusted for body mass index. Abbreviations: RS, Rotterdam Study; T2D, type 2 diabetes.

**Supplementary Table 12.** Associations of the HBI score with prediabetes and T2D risk after excluding participants that developed prediabetes and T2D within the first three years of follow-up (Sensitivity analyses)^a^.

|  |  |  | Prediabetes risk  (n=5,899/986 events) | | T2D risk  (n=6,571/703 events) | |
| --- | --- | --- | --- | --- | --- | --- |
|  |  |  | HR | 95% CI | HR | 95% CI |
| Model 1^b^ |  |  | 1.01 | (0.96, 1.05) | 1.03 | (0.97, 1.08) |
| Model 2^c^ |  |  | 1.02 | (0.98, 1.07) | 1.02 | (0.97, 1.08) |
| Model 3^d^ |  |  | 1.02 | (0.97, 1.07) | 1.02 | (0.97, 1.08) |

^a^Effect estimates are hazard ratios (HR) for prediabetes or T2D risk with their corresponding 95% confidence intervals (95% CI) per 10 points increment in HBI score. Estimates are based on pooled results of three RS sub-cohorts. ^b^Adjusted for age, sex, total energy intake, and RS sub-cohort. ^c^Additionally adjusted for smoking status, educational level, physical activity, and diet quality score. ^d^Additionally adjusted for body mass index. Abbreviations: RS, Rotterdam Study; T2D, type 2 diabetes.

**Supplementary Table 13.** Associations of the HBI score with high glucose and high insulin^a^.

|  |  | High glucose  (n=6,618) | |  |  | High insulin  (n=6,462) | |
| --- | --- | --- | --- | --- | --- | --- | --- |
|  |  | ORs | 95% CI |  |  | ORs | 95% CI |
| Model 1^b^ |  | 0.95 | (0.92, 0.99) |  |  | 1.01 | (0.98, 1.05) |
| Model 2^c^ |  | 0.97 | (0.93, 1.01) |  |  | 1.01 | (0.98, 1.05) |
| Model 3^d^ |  | 0.96 | (0.92, 1.00) |  |  | 1.00 | (0.96, 1.04) |

^a^Effect estimates are odds ratios (ORs) from logistic regression for high fasting glucose (≥5.55 mmol/L in fasting conditions) or high fasting insulin (≥60 pmol/L> mmol/L / no) levels at baseline, with their corresponding 95% confidence intervals (95% CI) per 10 points increment in HBI score. Estimates are based on pooled results of three RS sub-cohorts. ^b^Adjusted for age, sex, total energy intake, and RS sub-cohort. ^c^Additionally adjusted for smoking status, educational level, physical activity, and diet quality score. ^d^Additionally adjusted for body mass index. Abbreviations: Abbreviations: RS, Rotterdam Study.
